# Access to safe abortion is a fundamental human right

**DOI:** 10.1016/j.eclinm.2022.101426

**Published:** 2022-04-25

**Authors:** eClinicalMedicine

Abortion is a common medical or surgical intervention used to terminate pregnancy. Although a controversial and widely debated topic, approximately 73 million induced abortions occur worldwide each year, with 29% of all pregnancies and over 60% of unintended pregnancies ending in abortion. Abortions are considered safe if they are carried out using a method recommended by WHO, appropriate to the gestational age, and by someone with the necessary skills. Medical and surgical abortions can be safely managed by a trained health worker at a health-care facility. Medical abortions can also be safely self-managed outside of a health-care facility during the first 12 weeks of pregnancy. Global estimates suggest that approximately 45% of abortions are unsafe, defined as a procedure for termination delivered by persons without the necessary skills or in an environment not in conformity with minimal medical standards, or both. Wide disparities in the prevalence of unsafe abortions exist between high-income (12•5%) and low-income and middle-income (49•5%) countries or by the level of restriction of abortion laws. Unsafe abortions account for 4•7–13•2% of maternal deaths each year, with many more individuals experiencing other physical health complications, such as infection, haemorrhage, or uterine perforation, or psychological consequences, such as depression, anxiety, and eating disorders. People with unintended pregnancies often seek unsafe abortions due to legal restrictions, stigma or discrimination, financial barriers, or limits in access to safe family planning services.

In March 2022, WHO released new guidelines on the delivery of comprehensive abortion care, with recommendations covering three main domains: law and policy, clinical services, and service delivery. They recommend full decriminalisation of abortion, and that abortion be made available on request of the pregnant person. WHO advises against laws and other regulations that restrict abortion by grounds, calling for the formulation or revision of existing grounds in accordance with international human rights law until grounds-based approaches are removed. In terms of best practices and service delivery, WHO provides new recommendations for pain management in surgical and medical abortions, and for the option of telemedicine as an alternative to in-person interactions to deliver medical abortion care for self-managed abortions. These pivotal evidence-based recommendations and best practices recognise the needs of pregnant individuals across the abortion care pathway, calling for the removal of unnecessary policy barriers and providing clear guidance to health-care providers for the delivery of safe abortion care.

Although access to safe abortion worldwide is improving, legal barriers and stigma create wide disparities between countries. Over the last 25 years, at least 50 countries have liberalised their laws to improve access to abortion care; however, 90 million women of reproductive age still live in countries in which abortion is prohibited altogether (eg, in Egypt, Iraq, and the Philippines). Even in countries where abortion is lawful, policies vary widely, including whether it is regulated through criminal law. These legal constraints, together with other cultural and religious factors fuel abortion stigma, which can lead to feelings of shame, secrecy, and psychological distress over time in those who have sought an abortion, as well as marginalisation of abortion providers. As of February 2019, of 158 countries where abortion is lawful at the pregnant person's request, 28 (18%) restrict access to abortion-related information, 12 (8%) require spousal consent, and 56 (35%) permit service providers to conscientiously object provision of abortion care. In addition, constraints on gestational age limits, waiting periods, and lack of trained or willing providers, lead some pregnant individuals to travel to other countries with fewer restrictions to seek an abortion.

Certain populations can also face particularly difficult barriers to abortion care. People assigned female sex at birth but who do not conform to the gender binary (gender-expansive, non-binary, or transgender people) also encounter deterrents to abortion care caused by gendered health-care environments, misgendering, high financial costs, or discrimination. In a 2019 survey of individuals who do not conform to the gender binary, most respondents reported preferring a medication abortion rather than a surgical abortion, because they viewed this method as the least invasive and more private approach. Respondents also most frequently recommended that intake forms and health-care providers adopt gender-neutral or gender-affirming language to improve the quality and accessibility of abortion care.

In addition, despite the right to health under international law, migrants, asylum seekers, and displaced persons also encounter restrictive or no access to sexual and reproductive health services, or they are reluctant to exercise their right to available abortion care due to stigma, fear of deportation, or discrimination. Some migrant, refugee, or asylum-seeking women are at greater risk of sexual violence or sexual exploitation, thus increasing the risk of unwanted pregnancy. Despite this increased need for sexual and reproductive health care, these individuals encounter interpersonal, health-system, and sociocultural barriers preventing access to timely care.

It is clear that much work needs to be done to address the existing inequalities and stigma worldwide. Abortions occur regardless of the level of restriction. As such, a pregnant person's autonomous right to receive safe abortion care, should they decide to exercise it, must be respected. The morbidity and mortality caused by unsafe abortions can be avoided if necessary steps to decriminalise and destigmatise abortion are taken, and health-care systems provide universally accessible safe abortion care.

*eClinicalMedicine*

